# Improved detection of lipidated Atg8a by immunoblotting in *Drosophila melanogaster* cells and tissues enables precise investigation of Atg8a flux and its termination

**DOI:** 10.1080/15548627.2025.2508551

**Published:** 2025-06-19

**Authors:** Siri Andresen, Amani Al Outa, Miriam Formica, Jorrit Enserink, Helene Knævelsrud

**Affiliations:** a Department of Molecular Medicine, Institute of Basic Medical Sciences, University of Oslo, Oslo, Norway; b Centre for Cancer Cell Reprogramming, Institute of Clinical Medicine, Faculty of Medicine, University of Oslo, Oslo, Norway; c Department of Molecular Cell Biology, Institute for Cancer Research, Oslo University Hospital, Oslo, Norway; d Section for Biochemistry and Molecular Biology, The Department of Biosciences, Faculty of Mathematics and Natural Sciences, University of Oslo, Oslo, Norway

**Keywords:** Atg4, Atg8a, autophagic flux, macroautophagy, Drosophila, N-ethylmaleimide (NEM)

## Abstract

Macroautophagy/autophagy is an essential intracellular catabolic process for maintaining cellular homeostasis. In *Drosophila melanogaster*, Atg8a lipidation serves as a key marker for autophagy, yet traditional methods often fail to effectively detect its lipidated state. To overcome this limitation, we developed a refined approach that employs N-ethylmaleimide (NEM) to inhibit Atg4, thereby preserving Atg8a lipidation during sample preparation both *in vitro* and *in vivo*. We determined the optimal concentration of the autophagic inhibitors bafilomycin A_1_ (BafA1) and chloroquine (CQ) required for inhibition of autolysosomal degradation. Furthermore, we investigated the effects of prolonged nutrient deprivation on autophagic flux and TORC1 signaling. Our findings not only validate the effectiveness of this new approach to monitor lipidation of Atg8a but also provide insights into selection of autolysosomal inhibitors and nutrient-dependent regulatory roles of TORC1 in *Drosophila*.

## Introduction

Macroautophagy (hereafter referred to as autophagy) is an essential intracellular catabolic process that plays a critical role in maintaining cellular homeostasis [1]. This process is instrumental in the degradation of misfolded proteins, organelles, and pathogens, and it enables cells to adapt to metabolic stresses by facilitating the turnover of macromolecules. Autophagy is highly conserved across all eukaryotic organisms and has been extensively studied over the past few decades to understand its molecular mechanisms and roles in diverse physiological and pathological conditions. In this context, *Drosophila melanogaster* is a valuable model organism, due to its established relevance in cell biology and autophagy research [2].

The autophagic pathway is characterized by the formation of autophagosomes through the expansion of a membrane structure known as the phagophore [3]. These autophagosomes subsequently undergo fusion with lysosomes, resulting in the formation of autolysosomes where the enclosed cargoes are degraded and recycled. Central to this degradative process is the vacuolar-type H^+^-translocating ATPase (V-ATPase), a highly conserved proton pump that establishes and maintains the acidic environment within lysosomes in an ATP-dependent manner [4]. The V-ATPase is therefore crucial for lysosome-mediated degradation, as well as membrane trafficking, cargo sorting and signal transduction processes [5–8]. Bafilomycin A_1_ (BafA1) inhibits the V-ATPase by blocking proton translocation, thus resulting in elevated lysosomal pH which compromises lysosomal degradation efficiency [9–11]. BafA1 also inhibits the fusion between autophagosomes and lysosomes, a process that recent investigations have demonstrated to be independent of V-ATPase-mediated acidification [4]. Chloroquine (CQ) is another compound that can affect autophagy by elevating lysosomal pH through deprotonation upon its entry into the lysosomal lumen, thereby inhibiting lysosomal degradation [12]. However, recent studies have suggested that its primary mode of action may involve impairing autophagosome-lysosome fusion rather than altering lysosomal acidity [13].

Autophagy is a highly dynamic process that is tightly regulated through various mechanisms [14]. The Target of Rapamycin kinase complex 1 (TORC1) is a well characterized kinase complex that is known to negatively regulate autophagy. TORC1 functions as a downstream effector that senses nutrients and coordinates cell growth and metabolism through various signaling pathways. Under nutrient-rich conditions, TORC1 is activated by intracellular amino acids and growth factors, resulting in the phosphorylation and inhibition of core autophagy proteins. Furthermore, TORC1 suppresses autophagy by inhibiting transcription factors that are controlling expression of genes essential for lysosomal biogenesis and the autophagic machinery [15–18]. TORC1 also promotes various anabolic processes through the phosphorylation of key effectors such as RPS6KB/p70 S6 kinase/S6k, which subsequently facilitate mRNA translation [19–21], lipid synthesis [22], and nucleotide synthesis [23,24]. However, upon starvation, TORC1 is inhibited, resulting in autophagy initiation. In contrast to the initiation of autophagy, the mechanisms that regulate the termination of autophagy are not well-characterized [25]. One proposed mechanism involves the reactivation of TORC1, which can detect the increased availability of amino acids following lysosomal degradation by localizing to the lysosome surface [26–30]. This reactivation of TORC1 may then contribute to terminating autophagy.

The autophagy pathway requires a core machinery of Atg (autophagy related) proteins [31]. The nucleation and expansion of autophagic membranes are facilitated by multiple multi-subunit complexes, including the Atg1 complex, the class III phosphatidylinositol 3-kinase complex, the Atg9-Atg2-Atg18 complex, and the ubiquitin-like proteins Atg12 and Atg8a along with their conjugation systems. These two ubiquitin-like conjugation systems are responsible for mediating Atg8a lipidation onto the phagophore membrane. Prior to lipidation, the cysteine protease Atg4 cleaves Atg8a exposing a glycine at its C terminus, which enables the E1-like enzyme Atg7 to interact with Atg8a [32]. Following this, Atg8a is transferred to the E2-like enzyme Atg3 and then to phosphatidylethanolamine (PE). The transfer of Atg8a from Atg3 to PE is facilitated in part by the final product of the second conjugation system, the Atg12–Atg5-Atg16 complex. Following the expansion phase, the phagophore undergoes a sealing process to create the autophagosome [33]. During this phase, Atg4 catalyzes the cleavage between the C-terminal carboxyl group of Atg8a and the amino group of PE on the outer surface of the autophagosome, allowing Atg8a to be reused in subsequent conjugation reactions [34].

The role of Atg8a in autophagy extends beyond its function in autophagosome biogenesis. It also plays a crucial part in the cargo recruitment for autophagy, facilitated by interaction with specific cargo receptors [1]. In mammals, the polyubiquitin-binding protein SQSTM1/p62 function as a multifunctional scaffold protein involved in various cellular processes, including the selective recognition and isolation of cargo destined for degradation via autophagy [35]. SQSTM1 binds to ubiquitinated proteins and acts as a cargo receptor by interacting with Atg8a, thereby facilitating the autophagic degradation of ubiquitinated substrates [36,37]. *Drosophila* has one SQSTM1 homolog, ref(2)P (refractory to Sigma P) which shares similar functional motifs, including a C-terminal ubiquitin-associated domain [38,39].

Atg8a belongs to the family of ubiquitin-like proteins, including the MAP1LC3/LC3- and GABARAP-subfamilies in mammals [40]. *Drosophila* has two Atg8 homologs: Atg8a and Atg8b, with Atg8a being the primary focus due to its ubiquitous expression and functional relevance in autophagy, unlike the germline-specific Atg8b. Atg8a is more closely related to the mammalian GABARAP subfamily than to LC3 proteins. The lipidation of Atg8a is a critical step in autophagy and serves as a widely utilized marker for monitoring the process, due to its association with autophagic membranes. Western blot analysis is employed to differentiate between the unlipidated state of Atg8a (Atg8a-I) and the lipidated state (Atg8a-II) through SDS-PAGE [41]. Notably, Atg8a-II shows a faster electrophoretic mobility in SDS-PAGE gels compared to Atg8a-I, despite having a higher molecular mass. This increased migration is likely a consequence of increased hydrophobicity compared to the unconjugated form [42]. Effective separation between the two bands of Atg8a-I and Atg8a-II can help evaluate autophagy whereby increased levels of Atg8a-II protein compared to a loading control is used as a proxy for an increased number of autophagosomes [43–46]. In the mammalian system, an increase in LC3-II in the presence of lysosomal inhibitors such as BafA1 indicates increased cargo sequestration and autophagy induction. For LC3 flux assessment, LC3-II levels in the presence of BafA1 plus the treatment in question should be compared to LC3-II levels observed upon single treatment with BafA1 or the treatment in question alone. When the effect on LC3-II is additive, an increase in autophagic flux by the treatment in question can be inferred. A block in autophagic flux can be inferred if treatment alone increases LC3-II levels, but no further increase is observed by the treatment combined with BafA1 [42]. Despite accurate investigation of mammalian LC3, traditional western blotting protocols in *Drosophila* samples have proven inadequate, often failing to display the lipidated state of Atg8a.

Given these challenges, there is a pressing need for a refined method that can accurately detect lipidated Atg8a by immunoblotting in *Drosophila*. To address this need, we introduce an optimized approach that incorporates N-ethylmaleimide (NEM), which acts as a cysteine protease inhibitor [47,48], effectively inhibiting Atg4 and thereby preserving the lipidated state of Atg8a during sample collection and preparation. Here we demonstrate its efficacy both *in vitro* and *in vivo Drosophila* models, through rigorous experimentation and comparison with the existing approach. Utilizing this optimized method, we determined the optimal concentration of BafA1 and CQ for saturated inhibition of autolysosomal degradation in Schneider 2 R+ (S2R+) cells and *Drosophila* fat body tissue. Furthermore, we investigated the impact of starvation on Atg8a lipidation, autophagic flux, and TOR-signaling in S2R+ cells. Our results not only validate the effectiveness of this new approach but also provide insights into the selection of autolysosomal inhibitors and the regulation of autophagy by nutritional status, paving the way for more accurate and insightful autophagy research using this model organism.

## Results

### Lipidation of Atg8a is difficult to detect using standard conditions for protein lysis

To characterize the autophagic activity in S2R+ cells, we aimed to evaluate lipidated Atg8a levels by immunoblotting. We exposed S2R+ cells to conditions that are known to either induce autophagy (starvation for 4 h in serum- and amino acid-free medium) or inhibit autophagy (complete, nutrient-rich medium). To further probe the activity of autophagy, we employed the V-ATPase inhibitor BafA1. This pharmacological approach prevents Atg8a-II turnover, thus facilitating the quantification of total Atg8a-II accumulated during the drug treatment. BafA1 also prevents lysosomal ref(2)P degradation, thereby allowing evaluation of ref(2)P turnover by autophagy.

**Figure 1.**
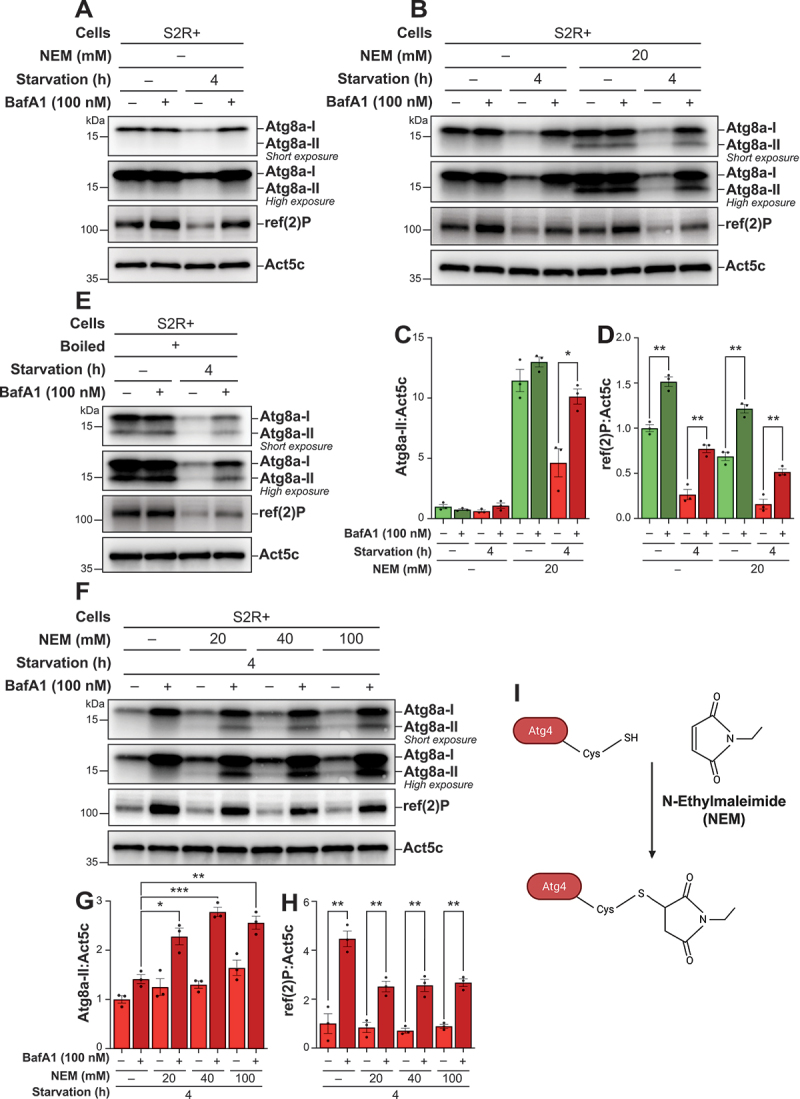
Incorporating N-ethylmaleimide (NEM) into the lysis buffer facilitates the detection of lipidated Atg8a by immunoblotting in S2R+ cells. (A, B, E, F) immunoblots of Atg8a at low (upper blot) and high (lower blot) exposure times and of ref(2)P. ACTB/β-actin/Act5C was used as a loading control. The cells were maintained in nutrient-rich medium or subjected to starvation for 4 h, in the presence of vehicle (DMSO) or 100 nM bafilomycin A_1_ (BafA1). (B, F) samples lysed with a lysis buffer containing an increasing concentration of NEM are compared to those lysed without. (E) S2R+ cells that were immediately boiled post-lysis, without the presence of NEM in the lysis buffer. (C, D, G, H) quantification of Atg8a-II and ref(2)P levels from immunoblots of three biological replicates from S2R+. Error bars represent the standard error of the mean. An unpaired t-test was performed to compare each vehicle-treated to the corresponding BafA1-treated samples (C, D), or BafA1-treated samples without NEM to BafA1-treated samples with increasing concentrations of NEM post-lysis (G, H). Statistically significant differences are indicated: *p < 0.05, **p < 0.01, ***p < 0.001. (I) schematic representation of the irreversible inhibition mechanism of Atg4 by NEM.

To evaluate autophagic flux, we extracted proteins from S2R+ cells using standard lysis buffer. Upon Atg8a antibody incubation, we detected only one band corresponding to the expected molecular mass of endogenous Atg8a-I (16 kDa) (Figure 1A). Unexpectedly, despite the induction of autophagy through starvation and drug-mediated inhibition of autophagic flux, the anticipated band for Atg8a-II (14 kDa) was not detected. Notably, we observed a marked decrease in Atg8a-I levels after 4 h of starvation compared to the nutrient-rich condition, which was reversed upon BafA1 treatment. Similarly, levels of ref(2)P decreased upon starvation, and this decrease was partly blocked by BafA1 treatment. These observations indicated that the S2 cells responded to starvation by activating autophagy. However, the absence of detectable Atg8a-II suggested two possibilities: the absence of Atg8a lipidation under the tested conditions, or a limitation in the sample preparation technique that precluded the detection of such lipidation.

### Addition of NEM enables observation of lipidated Atg8a in S2R+ cells

To test these possibilities, we refined our experimental methodology to detect Atg8a lipidation in S2R+ cells. We hypothesized that the Atg4 cysteine protease, which hydrolyzes the bond between Atg8a and PE, might be responsible for rapidly delipidating Atg8a during sample preparation. To prevent this, we supplemented the lysis buffer with 20 mM NEM, which acts by irreversibly alkylating the cysteine residue in the active sites of cysteine proteases. NEM has previously been used to inhibit Atg4 and preserve the lipidated state of mammalian GABARAP proteins [49].

The addition of 20 mM NEM to the lysis buffer resulted in a clear presence of a lipidated Atg8a band, confirming that NEM effectively preserves Atg8a lipidation during sample processing (Figure 1B, C). We then assessed autophagic flux under varying nutritional conditions by comparing Atg8a-II levels in cells maintained in nutrient-rich and starved states, both with and without BafA1 treatment. We found that Atg8a lipidation was high under nutrient-rich conditions, but its levels did not significantly increase with BafA1 treatment. In contrast, Atg8a-II levels were low under starvation, and increased significantly upon BafA1 treatment. These results indicate that NEM preserves Atg8a lipidation during lysis and that autophagic flux in S2R+ cells is minimal under nutrient-rich conditions but increases significantly during starvation.

Similarly, levels of ref(2)P were high under nutrient-rich conditions and decreased during starvation, an effect that was also partly blocked by BafA1 treatment (Figure 1B, D). Notably, although overall ref(2)P levels were slightly decreased in samples lysed with NEM, the overall turnover of ref(2)P showed minimal difference between samples lysed with or without NEM in the lysis buffer, indicating that this ref(2)P turnover is not influenced by the block of cysteine protease activity during lysis.

To ensure that NEM did not cause unintended effects on Atg8a-II levels, we performed an alternative protocol by subjecting the samples to immediate boiling in a NuPage sample buffer containing DL-dithiothreitol (DTT). This procedure rapidly denatures proteins and inactivates proteases, thereby allowing for the detection of lipidated Atg8a even in the absence of NEM. The patterns of Atg8a in the boiled samples closely resembled those treated with NEM (Figure 1E). However, the inclusion of SDS and DTT during the boiling process can complicate the quantification of proteins in subsequent analyses. In contrast, adding NEM directly to the lysis buffer does not affect downstream sample processing. From these findings, we concluded that the addition of NEM to the lysis buffer is an effective and reproducible strategy for detecting lipidated Atg8a via immunoblotting.

To ensure sufficient inhibition of Atg4 post-lysis, we subsequently explored a range of NEM concentrations, from 20 mM to 100 mM (Figure 1**F**). The addition of 40 mM NEM in the lysis buffer optimally facilitated the detection of lipidated Atg8a (Figure 1G). Elevating the concentration of NEM beyond this slightly decreased the levels of lipidated Atg8a, suggesting that 40 mM NEM is sufficient to effectively inhibit Atg4 and allow robust detection of Atg8a-II.

### Overexpressed fluorescently labeled Atg8a inhibits the detection of endogenous lipidation of Atg8a in S2 cells

**Figure 2.**
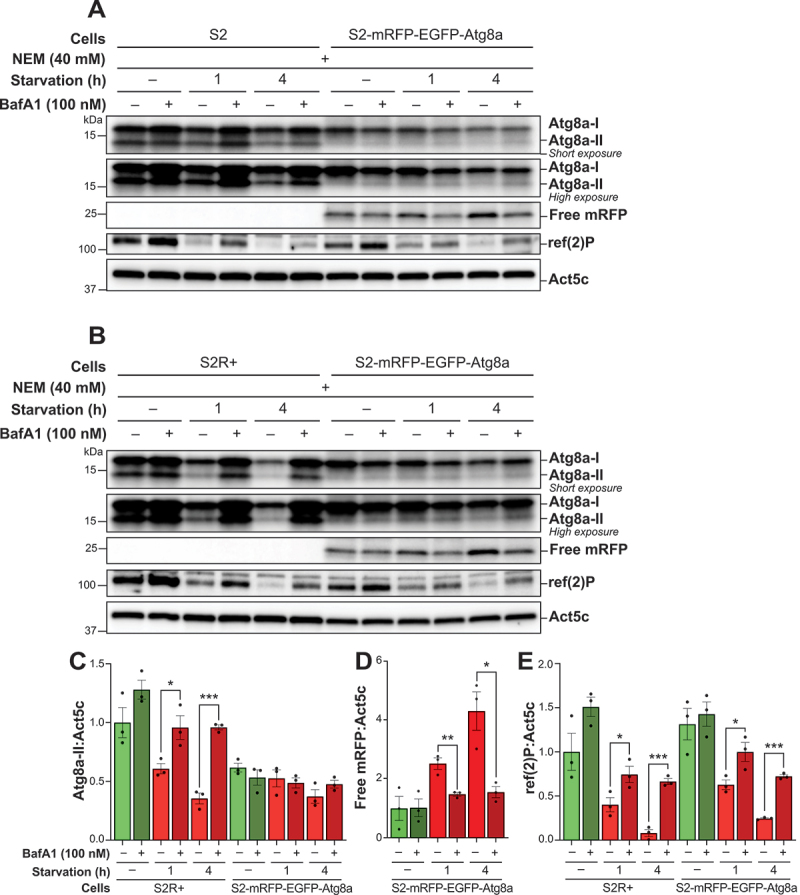
Overexpressed fluorescently labeled Atg8a inhibits the detection of endogenous lipidation of Atg8a in S2 cells. (A, B) immunoblots of Atg8a at low (upper blot) and high (lower blot) exposure times and of mRFP. ACTB/β-actin/Act5C was used as a loading control. S2 cells (A), S2R+ cells (B) and S2 cells expressing mRFP-EGFP-Atg8a were maintained in nutrient-rich medium or subjected to starvation for 1 h or 4 h, in the presence of vehicle (DMSO) or of 100 nM BafA1. All samples were lysed with a lysis buffer containing NEM. (C, D, E) quantification of Atg8a-II, free mRFP and ref(2)P levels from immunoblots of three biological replicates from S2R+ and S2-mRFP-EGFP-Atg8a cells. Error bars represent the standard error of the mean. An unpaired t-test was performed to compare each vehicle-treated to the corresponding BafA1-treated sample. Statistically significant differences are indicated: *p < 0.05, **p < 0.01, ***p < 0.001.

To evaluate how overexpression of a fluorescently tagged version of Atg8a affects endogenous Atg8a, we conducted comparative immunoblotting analyses on *Drosophila* S2 cell variants [50]. In both standard S2 cells and the more adherent S2R+ sub-line, distinct bands for Atg8a-I and Atg8a-II were observed (Figure 2A,B, respectively). During starvation, both cell types showed a progressive decrease in Atg8a-II levels, which was significantly reversed upon treatment with BafA1, indicating Atg8a flux (Figure 2C). However, S2 cells overexpressing mRFP-EGFP-Atg8a exhibited only a slight reduction in Atg8a-I during starvation, with a weak Atg8a-II band showing no significant changes, either during starvation or after impairing lysosomal degradation by BafA1.

To assess by other means whether the S2 cells overexpressing mRFP-EGFP-Atg8a responded to starvation by increasing autophagy, we employed cleavage of mRFP-EGFP-Atg8a as a read-out for autophagic flux. Upon induction of autophagy and fusion of mRFP-EGFP-Atg8a-containing autophagosomes to lysosomes, the generation of free mRFP or EGFP within autolysosomes can be detected via immunoblotting [42]. Notably, a band corresponding to free mRFP (~25 kDa) was detected by immunoblotting in all the samples from this cell line, aligning with the predicted molecular mass of 27 kDa (Figure 2A,B). Longer starvation durations resulted in an increased mRFP band intensity, an effect that was blocked by BafA1 (Figure 2D). Similarly, ref(2)P levels decreased with increasing time of starvation, which was accompanied by a significant increase upon BafA1 treatment (Figure 2E). This pattern was consistent across both S2R+ and S2-mRFP-EGFP-Atg8a cells, with similar ref(2)P flux between the two cell lines.

Collectively, these observations confirm that S2 cells overexpressing mRFP-EGFP-Atg8a are capable of initiating autophagy in response to starvation, despite the lack of evident changes in endogenous Atg8a-II levels by immunoblotting. These results underscore the need for caution when evaluating the lipidation levels of endogenous Atg8a in S2 cell lines that overexpress fluorescently tagged versions of the protein.

### NEM enables observation of lipidated Atg8a in Drosophila fat body tissue

**Figure 3.**
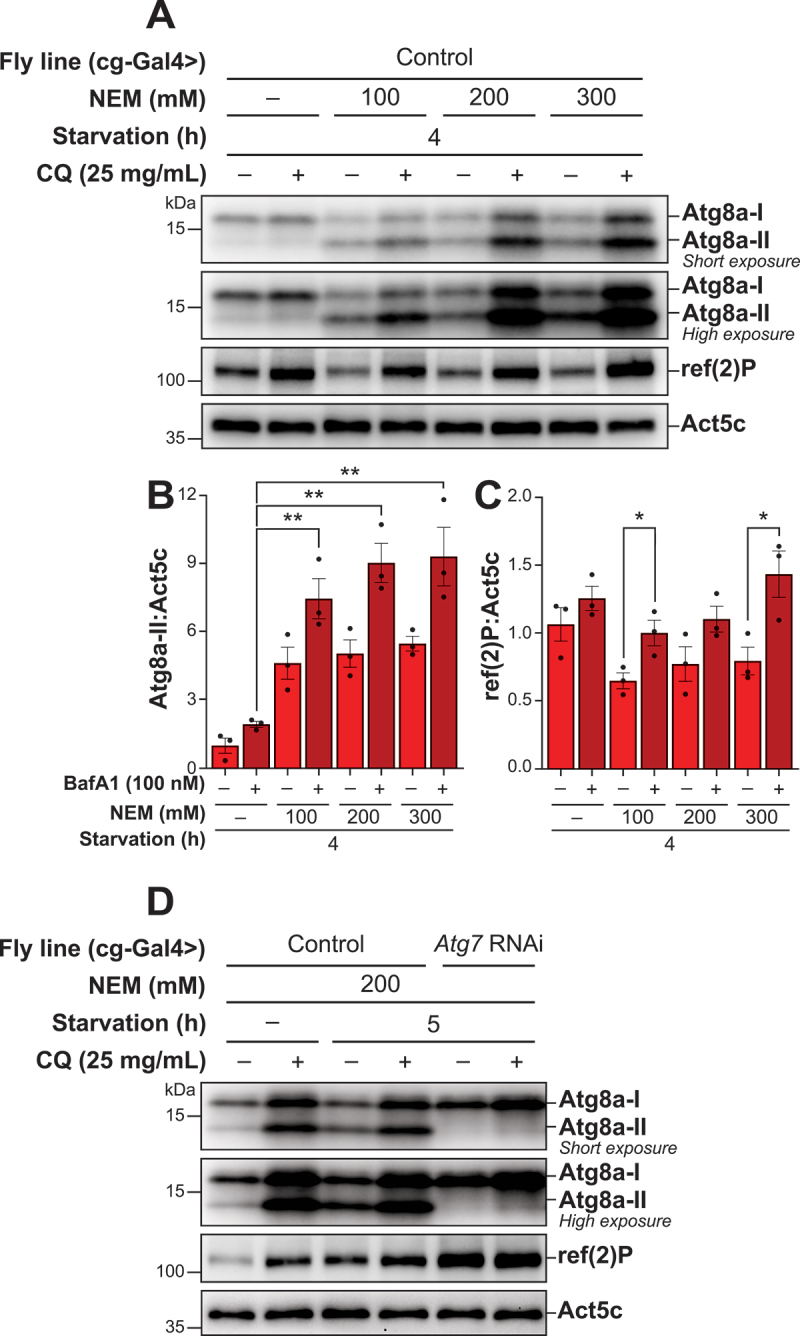
Incorporating NEM into the lysis buffer facilitates the detection of lipidated Atg8a by immunoblotting in *Drosophila* fat body tissue. (A, D) immunoblots of Atg8a with low (upper blot) and high (lower blot) exposure time and of ref(2)P in *Drosophila* fat body tissue. ACTB/β-actin/Act5C was used as a loading control. (A) *Drosophila* L3 larvae expressing control RNAi were starved for 4 h, treated with or without 25 mg/mL chloroquine (CQ) for the entire duration of the starvation period. Samples lysed with a lysis buffer containing an increasing concentration of NEM are compared to those lysed without. (B, C) quantification of Atg8a-II and ref(2)P levels from immunoblots of three biological replicates from *Drosophila* fat body tissue. Error bars represent the standard error of the mean. An unpaired t-test was performed to compare BafA1-treated samples without NEM to BafA1-treated samples with increasing concentrations of NEM post-lysis (B) or each vehicle-treated to the corresponding BafA1-treated sample (C). Statistically significant differences are indicated: **p < 0.01. (D) *Drosophila* L3 larvae expressing either control or *Atg7* RNAi, were kept on either standard fly food or starved in 20% sucrose for 5 h, treated with or without 25 mg/mL CQ for 5 h. All samples were lysed with a lysis buffer containing NEM.

Expanding upon the methodological improvement demonstrated in S2R+ cell experiments, we next aimed to extend this method to *in vivo* studies, specifically targeting the *Drosophila* larval fat body. The addition of NEM to the lysis buffer resulted in a clear presence of a lipidated Atg8a band, confirming that NEM also effectively preserves Atg8a lipidation for *in vivo* samples (Figure 3A). To identify the optimal concentration of NEM necessary for maintaining the lipidated state of Atg8a *in vivo*, we evaluated a concentration range from 100 mM to 300 mM NEM in starved samples treated with or without CQ. Our result indicated that a concentration of 200 mM NEM in the lysis buffer was sufficient to allow robust detection of Atg8a lipidation (Figure 3B). Notably, increasing the concentration of NEM beyond 200 mM resulted in only marginal increasing levels of lipidated Atg8a. The levels of ref(2)P increased upon CQ treatment, however the overall pattern appeared to be similar for samples lysed with or without NEM present in the lysis buffer (Figure 3C).

To confirm that NEM did not affect Atg8a-II lipidation *in vivo*, we utilized RNA interference (RNAi) to specifically downregulate Atg7, the E1-like enzyme in the Atg8a lipidation cascade, and subsequently evaluated the effect on autophagic flux during starvation. During starvation, we observed Atg8a lipidation which increased upon CQ treatment, indicating an increase in autophagic flux (Figure 3D). As expected, RNAi-mediated downregulation of Atg7 resulted in a complete absence of lipidated Atg8a, regardless of CQ treatment. Changes in the levels of ref(2)P followed the lipidation pattern of Atg8a during starvation, accompanied with a full block in ref(2)P degradation when Atg7 was downregulated. These observations underscore the effectiveness of NEM in preserving the lipidation state of Atg8a, while also accurately reflecting the cellular responses to autophagy modulation.

### NEM enables the precise determination of the optimal concentration of autolysosomal inhibitors in S2R+ cells and drosophila fat body tissue

**Figure 4.**
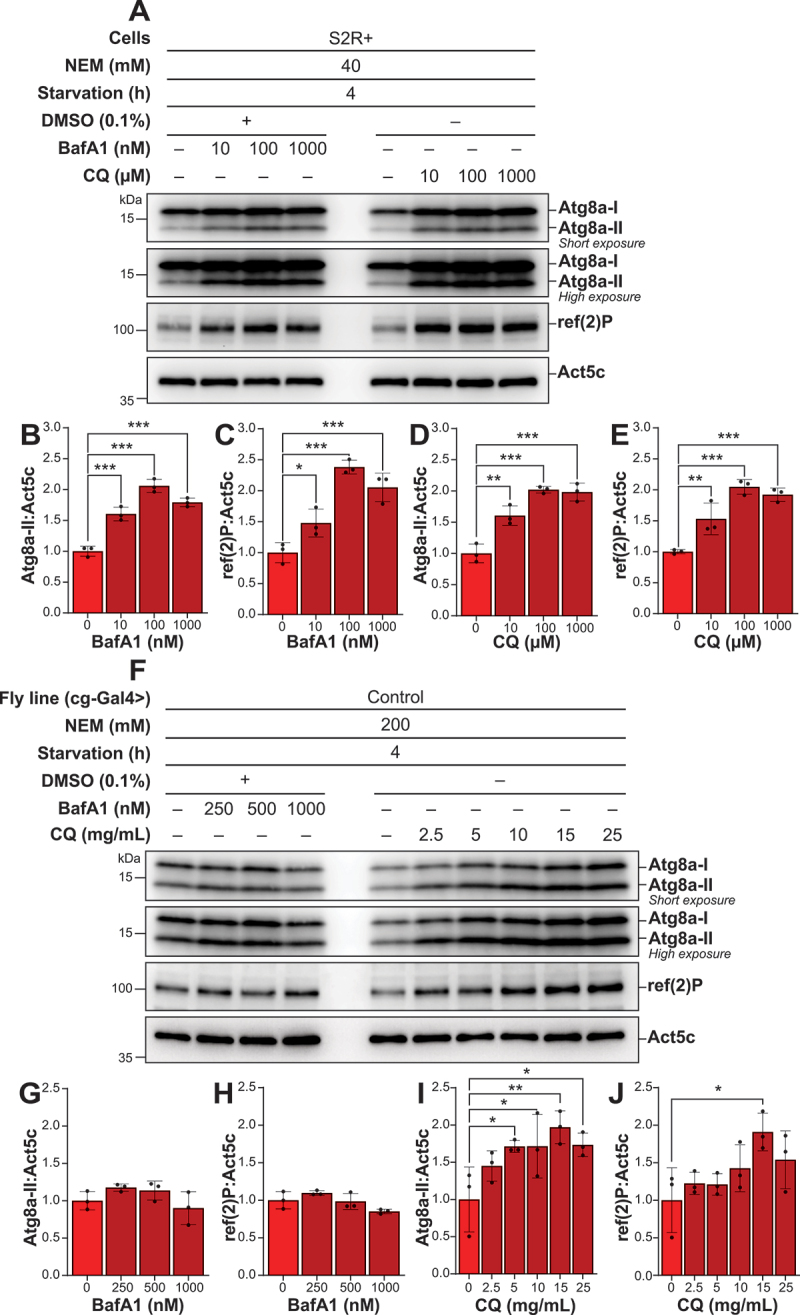
NEM enables the precise determination of the optimal concentration of autolysosomal inhibitors in S2R+ and *Drosophila* fat body tissue. (A, F) Representative immunoblots of Atg8a with low (upper blot) and high (lower blot) exposure time and of ref(2)P in S2R+ (A) and *Drosophila* fat body tissue (B). ACTB/β-actin/Act5C was used as a loading control. (A) S2R+ were subjected to starvation for 4 h, and either kept vehicle-treated or treated with an increasing concentration of BafA1 or CQ. All samples were lysed with a lysis buffer containing NEM. (F) *Drosophila* L3 larvae expressing control RNAi were starved in 20% sucrose for 4 h, and either kept vehicle-treated or treated with an increasing concentration of BafA1 and CQ. All samples were lysed with a lysis buffer containing NEM. (B-E, G-J) quantification of Atg8a-II and ref(2)P levels from immunoblots of three biological replicates from S2R+ (B-E) or *Drosophila* fat body tissue (G-J). Error bars represent the standard error of the mean. An ordinary one-way ANOVA was conducted followed by Dunnett’s multiple comparison test on all datasets, statistically significant differences are indicated: *p < 0.05, **p < 0.01, ***p < 0.001.

After optimization of a reliable protocol to detect Atg8a-II levels, we aimed to determine the optimal concentrations of the autolysosomal inhibitors BafA1 and CQ, in both S2R+ cells and *Drosophila* fat body tissue. We observed a significant increase in Atg8a-II levels in S2R+ cells treated with 100 nM BafA1 and 100 µM CQ, compared to the untreated controls, with no further increase upon treatment with higher concentration (Figure 4A, B, D). A similar pattern was detected for ref(2)P, with significantly high levels upon treatment with 100 nM BafA1 and 100 µM CQ (Figure 4A**, C, E**). In *Drosophila* fat body tissue, treatment with BafA1 resulted in minimal increases in both Atg8a-II and ref(2)P levels (Figure 4F–H). The slight increase observed at 100 nM BafA1 was reduced at the higher dose of 1000 nM, indicating that higher concentrations do not enhance autolysosomal inhibition. In contrast, CQ treatment displayed a clear dose-dependent increase in Atg8a-II levels, with a significant increased accumulation observed at 15 mg/mL (Figure 4F,I,J). This CQ concentration also led to the highest ref(2)P protein levels, whereas concentrations above 15 mg/mL did not further increase the levels of either Atg8a-II or ref(2)P.

In conclusion, our findings indicate that optimal concentrations for saturating lysosomal inhibition to measure autophagic flux in S2R+ cells are 100 nM BafA1 or 100 µM CQ, while in *Drosophila* fat body tissue CQ is most effective at concentrations of 15 mg/mL. These results validate the use of NEM for assessing changes in Atg8a lipidation and provide a reference for selecting appropriate inhibitor concentrations to study autophagy flux in different experimental settings.

### NEM affects S6k phosphorylation during lysis in S2R+ cells and drosophila fat body tissue

**Figure 5.**
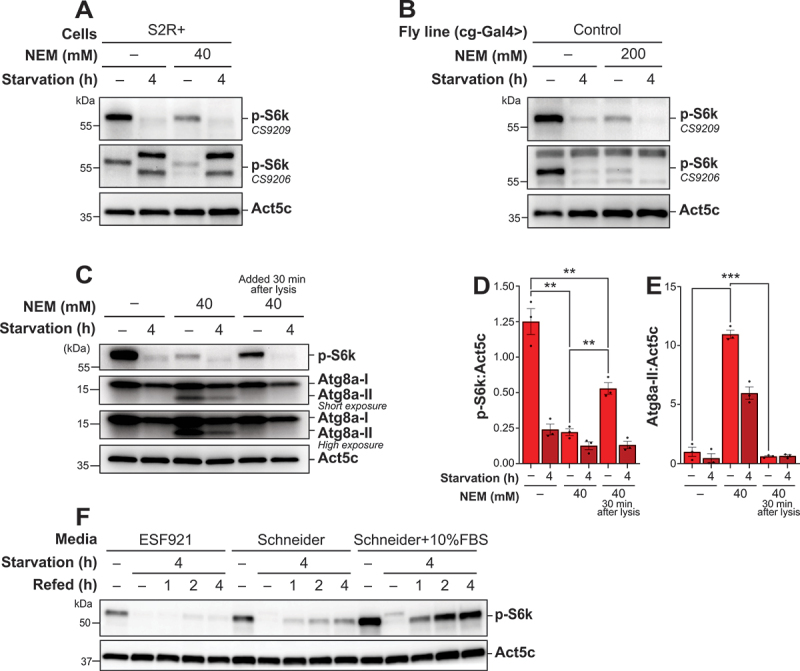
NEM affects S6k phosphorylation during lysis in S2R+ cells and *Drosophila* fat body tissue. (A, B, C, F) immunoblots of phospho-RPS6KB/p70 S6 Kinase/S6k (A, B, C, F) and Atg8a (C) with low (upper blot) and high (lower blot) exposure time. ACTB/β-actin/Act5C was used as a loading control. (A, B) the blots were probed using two different antibodies, CS9209 and CS9206, to detect phosphorylation at Thr398. (A, B, C) S2R+ cells (A, C) and *Drosophila* L3 larvae expressing control RNAi (B) were kept in nutrient-rich condition or starved in (1X) PBS supplemented with 2 mg/mL D(+)glucose (A, C) or in 20% sucrose (B) for 4 h. Samples were lysed with or without NEM in the lysis buffer. (D-E) quantification of p-S6k and Ag8a-II levels from immunoblots of three biological replicates from S2R+. Error bars represent the standard error of the mean. An ordinary one-way ANOVA was conducted followed by Dunnett’s multiple comparison test. Statistically significant differences compared to the fed sample immediately treated with NEM is indicated: **p < 0.01, ***p < 0.001. (F) S2-mRFP-EGFP-Atg8a cells were kept fed (either ESF921, Schneider, or Schneider medium supplemented with 10% FBS), starved in PBS (1X) supplemented with 2 mg/mL D(+)glucose for 4 h, or refed for 1 to 4 h in either ESF921, Schneider, or Schneider medium supplemented with 10% FBS. Samples were lysed without NEM added to the lysis buffer.

Under nutrient-rich conditions, an active TORC1 complex phosphorylates S6k at Thr398 in *Drosophila*, a marker we used to assess TORC1 activity indirectly. However, we found that incorporation of NEM into the lysis buffer, while facilitating lipidated Atg8a detection, reduced the detected phosphorylation of S6k, evidenced in both *in vitro* and *in vivo* samples (Figure 5A, B). We tested two S6k antibodies (CS9209 and CS9206) detecting S6k phosphorylation at the expected molecular weight in both S2R+ cells and *Drosophila* fat body tissue. The CS9209 antibody reliably detected a band corresponding in size to phosphorylated S6k, while the CS9206 antibody exhibited additional bands below and above the expected size. Consequently, CS9209 was selected for further use.

NEM preferentially reacts with thiol groups, forming a stable thioether bond by alkylating cysteine residues [47,48]. Given this reactivity, we hypothesized that NEM might interfere with the anti-phospho-S6k antibody’s epitope recognition by modifying cysteine residues near or within the epitope. Such modification could result in an apparent reduction in phosphorylation levels detected by western blotting or other techniques. However, sequence analysis of the region surrounding Thr398 revealed no cysteine residues near the phospho-site. Alternatively, the reduction in phosphorylation level could be attributed to an alteration in the activity or stability of proteins affecting S6k phosphorylation.

To explore these possibilities, we compared phosphorylation levels in samples lysed under three conditions: no NEM, 40 mM NEM, and the addition of 40 mM NEM to the lysis buffer 30 min after the initial lysis (Figure 5C). Consistent with our previous observations, the addition of NEM at the time of lysis enhanced Atg8a lipidation detection, but reduced S6k phosphorylation (Figure 5D**, E**). Interestingly, when NEM was added 30 min post-lysis, the detected S6k phosphorylation was still reduced, but much less than upon immediate NEM addition. This suggests that NEM’s impact on detection of phosphorylation is unlikely due to masking of the antibody epitope, as this effect would likely have been independent of the timing of NEM addition. Nevertheless, phosphorylation levels appeared reduced regardless of when NEM was introduced, and Atg8a-II was only detectable upon immediate presence of NEM in the lysis buffer. Given this observation, we proceeded to assess TORC1 activity without incorporating NEM in the lysis buffer during sample preparation, whereas Atg8a flux was assessed in samples where NEM was present during lysis. This observation highlights the need for careful consideration when using NEM in experiments assessing post-translational modifications, such as phosphorylation.

To further refine our conditions for detection of TORC1 activation, we investigated the effects of various cell culture media on S6k phosphorylation. Enhanced phosphorylation of S6k was observed in samples cultured in Schneider’s medium supplemented with 10% heat-inactivated fetal bovine serum (FBS), compared to those without heat-inactivated FBS or those cultured in serum-free ESF921 medium, where phosphorylation was much lower (Figure 5F). Phosphorylation of S6k also returned promptly after starvation by replenishing with complete Schneider’s medium, whereas this was also reduced in the other media conditions. These results suggest that the detection of TORC1 activity in these cells is easiest achieved by culturing in complete Schneider’s medium.

### Prolonged starvation leads to termination of autophagy without inducing reactivation of TORC1 activity in S2R+ cells

Having optimized TORC1 signaling activity and BafA1 concentrations, we next assessed autophagic flux in S2R+ cells over a time-course of starvation. Autophagic activity was monitored by comparing levels of Atg8a-II and ref(2)P in cells maintained in nutrient-rich conditions and exposed to acute serum and amino acid starvation for up to 6 h. BafA1 treatment was administered during the final hour before sample collection to enable the quantification of Atg8a-II and ref(2)P accumulation at hourly intervals throughout the starvation period.

**Figure 6.**
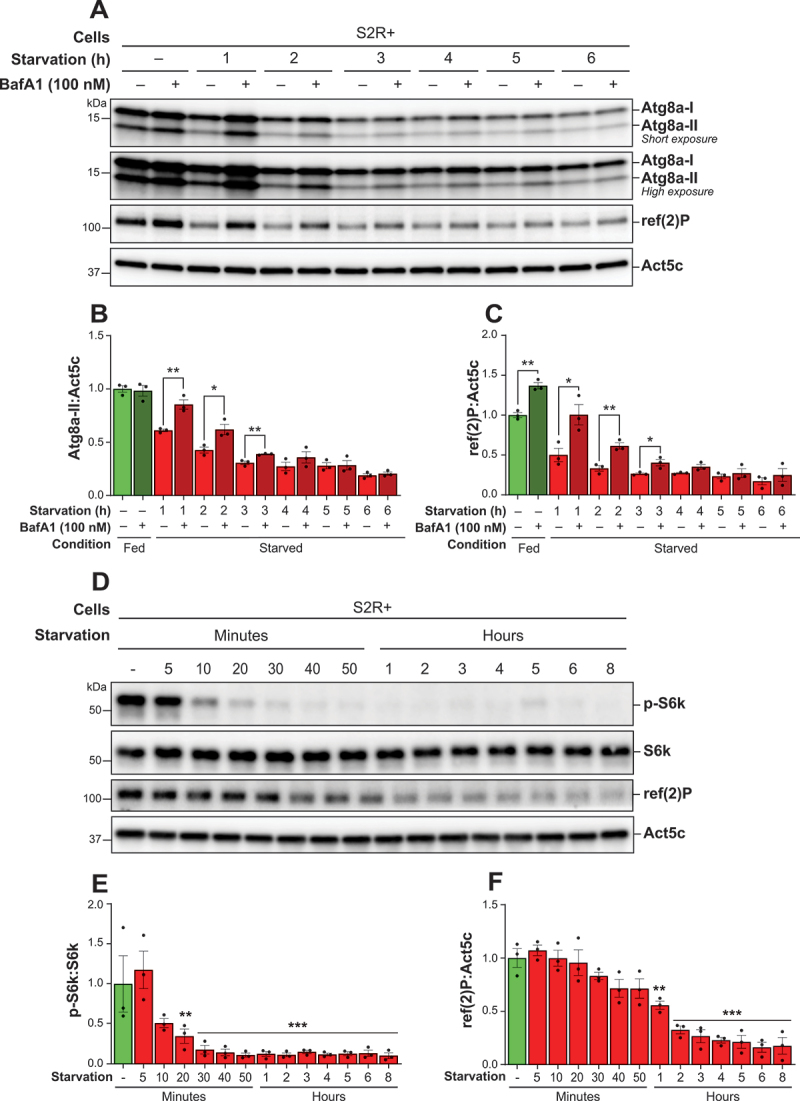
Prolonged starvation led to termination of autophagy, without inducing reactivation of TORC1 activity in S2R+ cells. (A) Representative immunoblots of Atg8a at low (upper blot) and high (lower blot) exposure times and of ref(2)P. S2R+ cells were maintained in nutrient-rich medium or subjected to starvation for 6 h. Where indicated, BafA1 was added during the last hour of starvation. All samples were lysed with a lysis buffer containing NEM. ACTB/β-actin/Act5C was used as a loading control. (B, C) quantification of Atg8a-II and ref(2)P levels from immunoblots of three biological replicates from S2R+. An unpaired t-test was performed to compare each vehicle-treated to the corresponding BafA1-treated sample. Error bars represent the standard error of the mean. Statistically significant differences are indicated: *p < 0.05, **p < 0.01. (D) Representative immunoblots showing phospho-RPS6KB/p70 S6 kinase/S6k and ref(2)P from S2R+ cells that were kept in nutrient-rich medium or starved up to 8 h. All samples were lysed without NEM in the lysis buffer. ACTB/β-actin/Act5C and RPS6KB/p70 S6 kinase/S6k were used as a loading control. (E, F) quantification of p-S6k and ref(2)P levels from immunoblots of three biological replicates from S2R+ cells. Error bars represent the standard error of the mean. An ordinary one-way ANOVA was conducted followed by Dunnett’s multiple comparison test. Statistically significant differences compared to fed are indicated: **p < 0.01, ***p < 0.001.

Atg8a-II levels were high in cells maintained in nutrient-rich conditions, with no significant alteration following BafA1 treatment, suggesting robust Atg8a lipidation with low Atg8a flux (Figure 6B). However, a substantial decrease in Atg8a-II levels was observed within the first 3 h of starvation, accompanied by a significant increase upon BafA1 treatment, indicating heightened autophagic flux during this period. Subsequently, Atg8a-II levels continued to decrease after 3 h of starvation and remained low throughout the 6 h starvation period, with little difference following BafA1-treatment, suggesting low flux. Similarly, ref(2)P levels were moderately elevated under fed conditions and significantly enhanced upon BafA1 treatment (Figure 6C). A notable reduction in ref(2)P was observed during the 3 h of starvation, accompanied by a significant increase in the BafA1-treated samples. Thereafter, ref(2)P levels remained low, with no differences between BafA1 treated and untreated samples up to 6 h of starvation. These patterns indicate that in S2R+ cells the autophagic flux is highest during the initial 3 h of starvation, but diminishes with persistent starvation.

Because TORC1 activity is known to negatively regulate autophagy, we asked whether this reduction in autophagic activity could be explained by reactivation of TORC1 in response to prolonged starvation. We observed that phosphorylation of S6k at Thr398 was high under nutrient-rich conditions (Figure 6E). However, with increased duration of starvation, the phosphorylation level of S6k decreased significantly after 20 min and persisted at very low levels throughout the entire 8-h starvation period. Similarly, ref(2)P levels gradually decreased with prolonged starvation, showing a significant reduction after 1 h compared to the nutrient-rich condition (Figure 6F). In conclusion, these results demonstrate that the increased autophagic flux in S2R+ cells during the initial hours of starvation is accompanied by suppressed TORC1 activity, but that TORC1 is not reactivated during the 8 h of starvation.

Overall, these findings validate the efficacy of NEM in preserving Atg8a lipidation with minimal variability across replicates. Thus, inclusion of NEM in the lysis buffer allows for robust detection of *Drosophila* Atg8a lipidation by immunoblotting and has potential as a valuable tool in autophagy research for generating reliable data. However, caution must be exercised when assessing various protein states using NEM in the lysis buffer, as demonstrated by the effect of NEM on the phosphorylation level of S6k post lysis.

## Discussion

The difficulties in detecting lipidated Atg8a in *Drosophila* S2R+ cells under standard lysis conditions highlighted the necessity for refining our methodology to accurately detect this autophagic marker. We here demonstrate the utility of including NEM to preserve Atg8a lipidation both *in vitro* and *in vivo*.

Based on this improved method, we could determine the optimal concentrations of the autolysosomal inhibitors BafA1 and CQ to monitor autophagic flux in S2R+ cells and *Drosophila* fat body. We observed that treating S2R+ cells with 100 nM BafA1 or 100 µM CQ significantly increased Atg8a-II and ref(2)P levels compared to untreated controls upon 4 h of starvation (Figure 4A–E). This is consistent with the concentrations frequently reported in the literature for autophagic flux inhibition in different cell types [42]. Notably, higher concentrations of these inhibitors led to a decrease in these autophagic markers, suggesting a saturation point where higher concentrations do not correspond with enhanced autophagic blockage but may instead induce cytotoxicity or nonspecific effects.

In *Drosophila* fat body tissue, CQ treatment demonstrated a clear dose-dependent increase in Atg8a-II and ref(2)P levels, which increased significantly at 15 mg/mL compared to the untreated control (Figure 4F,I,J). Interestingly, lower concentrations of CQ, such as 2.5 mg/mL and 3 mg/mL have frequently been utilized in previous studies to explore autophagic flux in *Drosophila* larvae across various tissues [51–55]. However, these concentrations of CQ did not saturate the inhibition of autophagic-lysosomal degradation in the current study. This discrepancy can be attributed to the different treatment durations employed, where the mentioned studies employed a longer treatment duration ranging from 12 to 72 h, compared to the 4-h treatment period used in this study. The lower CQ dosage at 2.5–3 mg/mL previously used may also reflect concerns noted in prior research where exposure to concentrations exceeding 10 mM were proven lethal to flies over a period of days [56]. However, within the 4-h treatment window in this study, no lethality was observed in larvae, even at CQ concentrations up to 50 mM. Still, another factor to consider when using autolysosomal inhibitors like CQ is the previously reported effect on overall autophagy levels [57–59].

In contrast to findings observed with CQ treatment, BafA1 demonstrated limited effectiveness in enhancing Atg8a-II and ref(2)P levels in *Drosophila* fat body tissue under conditions of 4-h starvation (Figure 4F–H). Even though both compounds function as late-stage inhibitors of autophagy, the observed difference between BafA1 and CQ may reflect differences in their molecular mechanisms of action. Differences in bioavailability between these compounds may also contribute to their varying efficacy in inhibiting lysosomal degradation in *Drosophila* fat body tissue. Monitoring autophagic flux *in vivo*, or in organ-specific contexts, remains a significant challenge and is currently one of the least developed areas in the field, with existing methodologies not entirely replicating those employed in cell culture studies [42]. To avoid the risks of reduced bioavailability and toxicity associated with compounds such as BafA1, an option is to analyze tissues *ex vivo*. Previous studies have developed an *ex vivo* culture system in which dissected *Drosophila* larval carcasses are incubated under various conditions [60]. In such settings, different concentrations of BafA1 have demonstrated the ability to impair autolysosome acidification in the *Drosophila* fat body tissue by causing the accumulation of Atg8a-positive puncta [4,61]. Another challenge when investigating the autophagic flux using Atg8a and ref(2)P with chemical perturbations, may be the difference in the speed of clearance of these autophagy markers in different systems, exemplified by our *in vitro* and *in vivo* results. Previous reports indicate that in mammals alterations in LC3 levels may be rapid while clearance of autophagic substrates like SQSTM1 May be slower hence necessitating investigation of their levels at later timepoints [42].

Our findings underscore the necessity for a careful selection of autophagy inhibitors when assessing autophagic flux, a choice that is highly dependent on the biological system and the nature of the research objectives. *In vitro*, BafA1 May offer certain advantages, particularly since CQ has been documented in mammalian cells to induce unconventional lipidation of LC3 on single-membrane intracellular compartments, an effect not observed with BafA1 [62–64]. Therefore, employing CQ may necessitate caution, especially when assays require differentiation between canonical and non-canonical autophagy pathways. The relevance of this unconventional lipidation to *Drosophila* models, however, remains inadequately investigated. Although BafA1 is a widely used autophagy inhibitor and may warrant high molecular functionality for certain research objectives, its poor toxicity profile has limited its use as a clinical intervention to block autophagy *in vivo*. In contrast, CQ has been widely used in clinical trials, presenting an advantage for *in vivo* experiments [65]. Moreover, CQ is its water soluble, thus eliminating concerns of toxicity or biological effects associated dimethyl sulfoxide (DMSO) as a solvent for BafA1.

NEM is widely employed for thiol group modification due to its specificity for cysteine residues [47,48]. In our experiments, the addition of NEM during cell lysis was observed to reduce the phosphorylation of S6k (Figure 5A–D). Previous studies have identified regulatory cysteine residues in various proteins, including transcription factors, kinases, phosphatases, and chaperones [66,67]. Notably, one study reported that NEM treatment, ranging from 30 min to 2 h, inhibited the phosphorylation of RPS6KB/p70 S6K downstream of PDGF-BB-stimulated AKT phosphorylation [68]. This finding demonstrates NEM’s ability to interfere with the phosphorylation cascade resulting in phosphorylation of S6k.

Importantly, while the previous study used NEM during active treatment over a 30 to 2 h window, our study applied NEM exclusively during the lysis step. Despite this difference in timing, we still observed a similar reduction in S6k phosphorylation. This indicates that NEM may rapidly alter the phosphorylation status of key proteins, even when applied post-treatment during lysis. The fact that these effects were observed solely during lysis suggests that upstream regulatory proteins may remain susceptible to NEM-induced modifications under lysis conditions. This hypothesis is further supported by previous reports [49] and our demonstration that the cysteine protease Atg4 retains its functionality under standard lysis conditions. Our results demonstrated that delaying the addition of NEM by 30 min post-lysis partially mitigates its inhibitory effect on phosphorylation detection (Figure 5D). However, by this stage, Atg4 has already delipidated Atg8a, preventing the detection of Atg8a-II by immunoblotting (Figure 5E).

The lack of knowledge surrounding the termination of autophagy represents a critical gap in the literature as proper termination of autophagy is crucial for maintaining cellular homeostasis. We aimed to investigate whether our improved protocol could be used to follow the autophagic flux upon prolonged starvation in *Drosophila* S2R+ cells as a means to show autophagy termination. By assessing Atg8a-II and ref(2)P accumulation at hourly intervals, we found that S2R+ cells exhibited the highest autophagic flux rates during the initial 2 h of starvation followed by an apparent termination of the autophagic flux, as evidenced by decreasing flux with time, after 3 to 4 h of starvation (Figure 6A–C). Previous studies support this observation, where prolonged nutrient deprivation was found to terminate autophagic activity in both yeast and rat kidney cells [26,27].

A proposed mechanism for the termination of autophagy involves the reactivation of TORC1. This reactivation is hypothesized to occur through the detection of increased amino acid availability resulting from lysosomal degradation, where TORC1 is believed to localize and reactivate on the lysosomal surface [26,30]. Similar to observations in both rat kidney cells, mouse embryonic fibroblasts, yeast and *Drosophila* fat body tissue [26–28,30], we observed a reduction in phosphorylation of S6k during the early phases of starvation (Figure 6D,E). However, contrary to the observed restoration of S6k phosphorylation after 6 h of starvation in rat kidney cells and *Drosophila* fat body tissue [26,28], our study did not observe a similar re-phosphorylation of S6k in S2R+ cells. This result aligns with findings from mouse embryonic fibroblasts [30], where autophagy, though essential, was not sufficient alone to replenish the amino acid pool required for TORC1 reactivation.

Late reactivation of TORC1 has been demonstrated in yeast to occur 12 to 24 h with persistent starvation [27], which could also be the case in S2R+ cells with extended starvation durations beyond the duration tested in our study. However, the fact that the autophagic flux appeared to be terminated within 3 to 4 h of starvation accompanied by lack of TORC1 reactivation implies the existence of potential TORC1-independent mechanism that terminate autophagy. Such mechanisms may involve proteasomal degradation of Atg proteins, as suggested in several studies in mammalian cells [69–71]. The findings from this study pave the way for further research into the molecular mechanisms terminating autophagy in *Drosophila*, offering potential parallels with mammalian systems.

It is important to note that our dependence on Atg8a and ref(2)P to assess autophagic flux might have constrained our ability to detect ongoing autophagic flux beyond 3 to 4 h of starvation. Notably, recent studies in mammalian cells have highlighted that bulk autophagy can proceed independently of LC3, even though the GABARAP subfamily was still found to be essential for autophagic sequestration [72]. Interestingly, *Drosophila* possesses only two homologs of Atg8a, namely Atg8a and Atg8b, with Atg8a being more closely related to the mammalian GABARAP subfamily rather than LC3 [40]. Therefore, the functional distinctions between GABARAP and LC3 subfamilies, and whether these findings translate to *Drosophila* models warrant further investigation. However, considering these findings, incorporating alternative assays that do not depend solely on autophagy markers but instead utilize more cargo-based assays would be valuable. Potential methodologies could include longed-live protein assay [73,74], lactate dehydrogenase sequestration assay [75], pH-sensitive fluorescent proteins such as Keima- or Rosella-based assays [76,77] and Halo-based processing assays [78].

## Materials and methods

### Cell culture

S2 cells (DGRC Stock 6; RRID:CVCL_TZ72), S2R+ cells (DGRC Stock 150; RRID:CVCL_Z831), and S2-mRFP-EGFP-Atg8a cells (DGRC Stock 290; RRID:CVCL_XF59) [79] were cultured in Schneider’s *Drosophila* Medium (GIBCO 21,720,001) supplemented with 10% heat-inactivated FBS (GIBCO, F7524) and 1% penicillin-streptomycin (GIBCO 15,140–122) (hereby referred to as complete, nutrient-rich medium) at 25°C. For selection, S2-mRFP-EGFP-Atg8a cells were maintained in 0.01 mg/mL Zeocin (Invitrogen, R25001). ESF921 Insect Cell Culture Medium (Expression Systems 500,302) was used in some experiments.

### Fly strains

Fly strains were kept and raised in bottles containing standard potato mash fly food: 27.3 g/liter dry yeast (LeSaffre 41,036), 32.7 g/liter dried potato powder (Hoff 24,458), 60 g/liter sucrose (NordicSugar AS 10,330), 0.73% agar (AS PALS 77,000), 0.2% Methyl 4-hydroxybenzoate (Sigma-Aldrich, H5501) dissolved in ethanol and 0.45% propionic acid (Sigma-Aldrich, P1386). Crosses were kept at 25°C, with egg deposition allowed for a period of 4 h, before being incubated for a total of 96 h at 25°C. *Drosophila* lines used in this study were provided by the Bloomington Drosophila Stock Center: UAS-RLuc RNAi (31603), cgGal4 (7011), UAS-Atg7 RNAi (27707).

### Treatment and inhibitors

For *in vitro* starvation experiments, S2 cells were acutely deprived of serum and amino acids, using 1X phosphate-buffered saline (PBS; Gibco 14,190–094) supplemented with 2 mg/mL D(+)glucose (Formedium, GLU03), while fed control were kept in complete, nutrient-rich medium.

For *in vivo* starvation experiments, staged third instar (L3) feeding larvae were washed and placed in 20% sucrose (Sigma-Aldrich, S7903) diluted in PBS. Fed larvae were kept on standard fly food with food colorant (Panduro 1,018,108–104), enabling the visual inspection of food in their gastrointestinal tracts.

To assess autophagic flux, autolysosomal degradation was inhibited using BafA1 (Enzo Life Sciences, BML-CM110-0100) dissolved in DMSO or CQ (Sigma-Aldrich, C6628) dissolved in water for the indicated times and concentrations. Equal volume of DMSO was used as vehicle control for BafA1-treated samples.

### Sample preparation and lysis

For *in vitro* studies, S2 cells were seeded in 6-well plates at 1 × 10^6^ cells/well and grown to 80% confluence overnight. Following this, various treatments were applied to each well at the indicated times and concentrations. At the end of the treatment period, the plates were transferred to ice and medium was aspirated from each well. To ensure thorough removal of all residual media, the wells were then washed twice with ice-cold PBS. After the final wash, the plates were tilted at a 90-degree angle, facilitating the removal of any remaining liquid. Cell lysates were prepared using ice cold RIPA Buffer (50 mM Tris-HCl, pH 7.4, 150 mM NaCl, 0.25% deoxycholic acid, 1% NP-40, and 1 mm EDTA; Millipore, 20–188), supplemented with phosphatase (Roche 04,906,837,001) and protease (Roche 05,056,489,001) inhibitors (herby referred to as RIPA lysis buffer) as well as freshly prepared NEM (Sigma-Aldrich, E3876). To prevent hydrolysis of the maleimide group, it is recommended to dissolve NEM immediately before use and dispose of excess reagents. For that reason, the desired concentration of NEM was added to a predetermined amount of lysis buffer for each experiment. Due to the small quantity required, a 0.1 M NEM stock solution dissolved in water was initially prepared. This stock solution was then diluted into a more concentrated lysis buffer to achieve the desired final concentration of NEM in a 1X lysis buffer, and then discarded after use. For instance, to obtain a final concentration of 40 mM NEM, 400 µL of 0.1 M NEM stock was combined with 600 µL of 1.67X lysis buffer to yield a 1X lysis buffer suitable for use. The total volume of the lysis buffer needed was calculated in advance, and the volume was adjusted accordingly.

Approximately 150 µL of lysis buffer supplemented with the desired concentration of NEM was added to each well, before cell scrapers were used to collect the lysate, which was then transferred to a 1.5-mL microcentrifuge tube. To ensure that the lipidated state of Atg8a was preserved, extra care was taken to pre-cool the 1.5-mL microcentrifuge tubes and cell scrapers, and to keep the samples on ice throughout the process.

For *in vivo* studies, *Drosophila* larvae were floated by adding 20% sucrose in PBS to each vial. They were then carefully transferred to a 24-well plate, containing 1 mL 20% sucrose. The larvae underwent three washes to remove food debris by carefully pipetting to ensure thorough mixing of the larvae before moving them into a new clean well. After the washing procedure, the larvae were transferred to new wells containing 1 mL of 20% sucrose solution in each, with a maximum of 15 larvae per well. Larvae destined for feeding were similarly floated in 20% sucrose, but then immediately transferred to a 24-well plate containing 1 mL of freshly prepared colored standard potato mash fly food in each well.

Following the treatment period, fat body from approximately 10 *Drosophila* larvae were dissected for each sample. To ensure that the lipidated state of Atg8a was preserved, precautions were taken to inhibit Atg4 as fast as possible during dissection. For that reason, to prepare for sample collection, multiple drops of PBS were placed evenly around a drop of lysis buffer centered in the middle on a dissection plate. The lysis buffer was supplemented with NEM, and approximately 50 µL was used per sample. As a higher concentration of NEM was used for *in vivo* studies, the desired concentration was dissolved directly in 1X lysis buffer, eliminating the need for a pre-made stock solution. However, at such a high concentration, NEM will precipitate if it is placed directly into ice. For that reason, the lysis buffer was rather positioned on top of the ice, ensuring that it remained sufficiently cool without inducing NEM precipitation.

Each larva was then dissected in an individual drop of PBS to ensure a clean dissection environment. The process was initiated by removing the posterior end with a tweezer, followed by inverting the larval carcass by clamping the tweezer on the head and rolling the posterior end onto the tweezer blade, thereby exposing the internal tissues. All tissues except the fat body were carefully removed. The strategic arrangement of the dissection plate allowed for the continuous transfer of the isolated fat bodies immediately to the adjacent drop of lysis buffer. During this step, extra care was taken to ensure that only the intended tissue was transferred, as any other tissue or contaminants could affect subsequent analyses. Once the dissection of the total amount of larvae for a given sample was complete, the drop of lysis buffer, now containing the fat bodies, was carefully transferred by pipetting into a 1.5-mL microcentrifuge tube for further processing.

The cell lysates were centrifuged for 15 min at 14,000 × g at 4°C before the supernatant was transferred to a new tube while the pellet was discarded. However, the *in vivo* samples required more preparation, and were first pestled for 5 to 10 s on ice using a pestle rotator (DWK Life Sciences 749,540–0000) before being centrifuged through a QIAshredder column (Qiagen 79,656) at 18,000 × g for 3 min. Next, the lysates were centrifuged once at 14,000 × g for 15 min at 4°C, before the supernatant was transferred into a new 1.5-mL microcentrifuge tube, while taking extra care not to transfer the pellet in the bottom or the fat lid on top. Finally, this cleared supernatant was centrifuged one last time for 15 min at 14,000 × g at 4°C, before the supernatant was transferred to a new tube while the pellet was discarded.

### Western blot

Supernatant was used for protein quantification using the bicinchoninic acid (BCA) Protein Assay Kit (Thermo Fisher 23,227). Protein extract was mixed with 4X sample buffer (Thermo Fisher, NP0008) and 10X 1 M DTT (Sigma-Aldrich, D0632) to a final concentration of 1X and boiled for 5 min at 95°C. The samples were separated by SDS-PAGE on 4–20% gradient gels (Bio-Rad, 567–1094, 567–1095). Proteins were then transferred onto LF PVDF membrane (Bio-Rad, 161–0374) using the semi-dry Trans-Blot® Turbo™ Transfer System (Bio-Rad 1,704,150). The membrane was air dried for 15 min before incubation with primary antibody and rotated overnight at 4°C. Next day, the membrane was washed three times for 10 min in Tris-buffered saline (TBS; Bio-Rad 10,026,938) with 0.1% Tween 20 (Sigma-Aldrich, P1379), and then incubated with the appropriate horseradish peroxidase (HRP)-conjugated secondary antibody in TBS-Tween 20 with 5% skim milk powder (Millipore 70,166) for 1 h at room temperature. The antibodies were detected by chemiluminescence, using SuperSignal West DURA Extended Duration Substrate (Thermo Fisher 11,593,440) and captured using ChemiDoc MP system (Bio-Rad). The immunoblots were quantified using Image Lab 6.1 Software.

### Antibodies

Primary antibodies and dilutions were: Rabbit anti-GABARAP+GABARAL1+ GABARAL2 1:1000 (Abcam, ab109364; to detect Atg8a), rabbit anti-ACTB/β-actin/Act5C 1:1000 (Abcam, ab8227), goat anti-mCherry, 1:500 (Acris, AB0040–200), rabbit anti-phospho-Drosophila S6k/p70 S6 kinase (Th398) 1:500 (Cell Signaling Technology, 9209), mouse anti-phospho-RPS6KB/p70 S6 kinase/S6k (Th389) 1:500 (Cell Signaling Technology, 9206), rabbit anti-ref(2)P 1:250 (Abcam, ab178440), guinea pig anti-S6k 1:10,000 (kindly provided by the Mary Lilly lab (NIH, Bethesda, Maryland) [80]). HRP-conjugated secondary antibodies and dilutions used were: anti-rabbit 1:5000 (Jackson, 111-035-144), anti-goat 1:5000 (Jackson, 705-035-147), anti-guinea pig 1:5000 (Jackson, 706-035-148), anti-mouse 1:5000 (Jackson, 115-035-003). A full list of the antibodies can be found in Table 1.

**Table 1.** Key resource table.

Reagent or Resource	Source	Identifiers
**Antibodies**		
Goat Anti-mCherry (1:500)	Acris Antibodies	AB0040 RRID:AB_2333093
Guinea pig anti-S6k (1:10.000)	Mary Lilly Lab	[80]
HRP-donkey-anti-goat (1:5000)	Jackson	705-035-147 RRID:AB_2313587
HRP-donkey-anti-guinea pig (1:5000)	Jackson	706-035-148 RRID:AB_2340447
HRP-goat-anti-mouse (1:5000)	Jackson	115-035-003 RRID:AB_10015289
HRP-goat-anti-rabbit (1:5000)	Jackson	111-035-144 RRID:AB_2307391
Mouse Anti-Phospho-RPS6KB/p70 S6 kinase/S6k (Thr389; 1:500)	Cell Signaling Technology	9206 RRID:AB_2285392
Rabbit Anti-GABARAP+GABARAPL1+GABARAPL2 (1:1000)	Abcam	ab109364 RRID:AB_10861928
Rabbit Anti-phospho-Drosophila S6k/p70 S6 kinase (Thr398; 1:500)	Cell Signaling Technology	9209 RRID:AB_2269804
Rabbit Anti-ref(2)P (1:250)	Abcam	ab178440 RRID:AB_293880
Rabbit Anti-ACTB/β-actin/Act5c (1:1000)	Abcam	ab8227 RRID:AB_2305186
**Chemicals**		
Bafilomycin A_1_	Enzo Life Sciences	BML-CM110-0100
Chloroquine diphosphate salt	Sigma-Aldrich	C6628
N-Ethylmaleimide	Sigma-Aldrich	E3876
PhosSTOP EASYpack	Roche	04906837001
Protease Inhibitor Cocktail	Roche	05056489001
Zeocin™ Selection Reagent	Invitrogen	R25001
**Media**		
ESF921 Insect Cell Culture Medium	Expression Systems	500302
Schneider’s Drosophila Medium	Gibco	21720001
**Experimental models**		
S2 cells	DGRC Stock 9	RRID:CVCL_TZ72
S2-mRFP-EGFP-Atg8a cells	DGRC Stock 290	RRID:CVCL_XF59
S2R+ cells	DGRC Stock 150	RRID:CVCL_Z831
cgGal4	BDSC Stock 7011	RRID:BDSC_7011
UAS-RLuc RNAi	BDSC Stock 31,603	RRID:BDSC_31603
UAS-Atg7 RNAi	BDSC Stock 27,707	RRID:BDSC_25896
**Instrument and software**		
Adobe Illustrator	Adobe	RRID:SCR_010279
ChemiDoc^TM^ MP Imaging System	Bio-Rad	RRID:SCR_019037
Fluostar OPTIMA	BMG LABTECH	
GraphPad Prism 9	GraphPad Software	RRID:SCR_002798
ImageLab 6.1	ImageLab Software	RRID:SCR_014210
Trans-Blot Turbo transfer system	Bio-Rad	

### Statistical analysis

Statistical analyses were conducted using GraphPad Prism version 10.2.0. Data normality was tested and generally confirmed, followed by either unpaired t-test to compare specific conditions or an ordinary one-way ANOVA test and Dunnett’s Multiple Comparison Test at a significance level of 0.05 (95% CI) to compare across all conditions. The results are presented as mean ± standard error of the mean, and p-values less than 0.05 were considered statistically significant (* *p* < 0.05, ** *p* < 0.01, *** *p* < 0.001).

## Conclusion

In summary, the protocol described here represents a reliable and widely applicable method to monitor lipidation of Atg8a in *Drosophila*, both *in vitro* and *in vivo*. Taking advantage of the developed method using NEM enabled consistent detection of lipidated Atg8a consolidating the relevance of *Drosophila* to investigate and characterize autophagy.

Protocol

## Sample preparation

### In vitro samples

#### Cell collection

- Gently pipette the cell suspension up and down (~10 times) in a T75 flask to detach the cells. Transfer to a 15-mL tube and centrifuge at 1000 × g for 3 min at 25°C.

#### Supernatant removal and cell resuspension

- Carefully aspirate and discard the supernatant without disturbing the cell pellet. Resuspend the pellet in 10 mL of Schneider’s *Drosophila* Medium (Gibco 21,720,001) supplemented with 10% heat-inactivated FBS (Sigma-Aldrich, F7524) and 1% penicillin-streptomycin (Gibco 15,140,122) (hereby referred to as complete, nutrient-rich medium).

#### Cell counting

- Mix 10 µL of cell suspension with 10 µL of 0.4% Trypan Blue. Load onto a counting chamber slide and use a cell counter to record the cell count.

#### Cell seeding and incubation

- Seed cells at a density of 1 × 10^6^ per well in 6-well plates (35-mm wells). Adjust volume to 1.5 mL per well. Incubate plates at 25°C overnight until 80% confluency.

**Notes**:

- Maintain sterile techniques throughout the experiment to prevent contamination.
- Ensure that the FBS is heat inactivated. Serum contains active components such as the complement system, which can adversely affect the growth and viability of *Drosophila* S2 cells. Heat inactivation is crucial to deactivate these heat-labile components, thereby ensuring optimal culture conditions.

### In vivo samples

#### Virgin collection and setting up crosses

- Collect virgin female *Drosophila* flies prior to the experimental setup day and house them in an incubator at 18°C.
- Set up crosses two days before the planned egg deposition by preparing the desired number of fly tubes based on the scale of the experiment. Fill the tubes with fly food composed of 27.3 g/liter dry yeast, 32.7 g/liter dried potato powder, 60 g/liter sucrose, 0.73% agar, 0.2% methyl 4-hydroxybenzoate (nipagin) dissolved in ethanol and 0.45% propionic acid (hereby referred to as standard potato mash fly food). Apply a small amount of yeast paste to the inside wall or the plug of each tube, to stimulate mating behavior.
- Add the desired genotypes in the ratio of 10 virgin females to 5 males in each fly tube. This ratio can be adjusted based on the specific genotypes to optimize the mating frequency. Then place the crosses in an incubator set at 25°C.

#### Egg deposition

- Transfer the crosses into the freshly prepared tubes and push the plug downwards to reduce the space available for flies, thereby facilitating more mating and egg-laying.
- Allow the flies to deposit eggs for a period of 4 h to synchronize the development of the larvae. Monitor the egg deposition closely and depending on the specific genotype and observed egg production rates, adjust the duration to ensure that a sufficient number of eggs are laid.
- To terminate the egg deposition, transfer the adults to new tubes, before storing both the egg collection tubes and the new fly tubes with adults at 25°C.

#### Move larvae

- Move larvae within 72 h after egg deposition.
- To facilitate the floating of larvae, add a 20% sucrose (Sigma-Aldrich, S7903) solution to the fly tube. Then transfer the 20% sucrose solution with the floating larvae into a Petri dish before carefully transferring larvae into new tubes using a tweezer (~15 larvae per tube).
- Place the newly prepared tubes with transferred larvae back at 25°C. Allow the larvae to develop undisturbed until they reach the desired L3 feeding stage, 96 h after egg deposition.

**Notes**:

- Consistent environmental and nutritional conditions are critical to ensure uniform larval development. It is therefore critical to move the larvae before the experiment, so that the larvae develop in a fly tube that is not overcrowded.
- Handle larvae and food media with care to prevent injury and stress to the larvae, which could affect experimental outcomes.

## Experimental Procedure

### In vitro samples

#### Starvation

- Wash cells twice with starvation medium, 1X PBS (Gibco 14,190–094) supplemented with 2 mg/mL glucose (Formedium, GLU03), before adding 1.5 mL to each well.
- For cells designated to remain fed, carefully aspirate any old media from the wells and add 1.5 mL of new complete nutrient-rich medium to each of these wells to maintain nutrient availability.

### In vivo samples

#### Starvation and feeding

- Transfer floating larvae into a 24-well plate designated for washing, with three or more wells pre-filled with 2 mL of the 20% sucrose solution.
- Conduct three gentle washes to cleanse the larvae of any debris, by pipetting the solution carefully up and down. After each wash, transfer the larvae into a new, clean well filled with fresh 20% sucrose solution, continuing the gentle mixing.
- After completing the washes, transfer the larvae to a new 24-well plate, where each well contains 1 mL of 20% sucrose solution (~15 larvae per well).
- For feeding larvae, transfer larvae directly into 24-well plates containing 1 mL standard fly food without additional washing steps in between. Depending on the experimental requirements, the potato mash fly food may be colored to facilitate the visual inspection of food within the larvae’s gastrointestinal tracts.

## Protein Extraction

### In vitro samples

#### Reagent preparation

**25X Protease inhibitor cocktail, stock solution**: Dissolve one protease inhibitor cocktail tablet (Roche 05,056,489,001) in 2 mL of mqH_2_O to achieve a 25X stock solution. Aliquot 400 µL and store at −20°C.

**1.67X RIPA lysis buffer, stock solution**: Mix 1 mL 10X RIPA (Millipore, 20–188), 400 µL 25X Protease Inhibitor, 4.6 mL of mqH_2_O as well as 1 tablet of Phosphatase Inhibitor Cocktail (Roche 04,906,837,001). Aliquot and store at −20°C.

**0.1 M NEM, stock solution**: Dissolve 0.031 g NEM (Sigma-Aldrich, E3876) (molecular weight = 125.13 g/mol) in 2.5 mL mqH_2_O.

**1X RIPA lysis buffer with 40 mM NEM, working solution**: For 10 wells, prepare 1000 µL total 1X lysis buffer. To reach a final concentration of 40 mM NEM, combine 400 µL of 0.1 M NEM stock solution with 600 µL of 1.67X RIPA buffer.

#### Cell lysis

- Wash the cells twice with ice-cold PBS on ice. Before tilting the 6-well plate nearly vertically for a few seconds to pool residual PBS at the bottom, then carefully remove all remaining liquid.
- Add 100 µL of pre-prepared, ice-cold 1X RIPA lysis buffer containing 40 mM NEM to each well.
- Using a cell scraper, scrape the cells of the dish and transfer the cell suspension into pre-cooled 1.5-mL microcentrifuge tubes.
- Centrifuge at 14,000 × g at 4°C for 15 min to pellet cell debris. Transfer the supernatant into a new pre-cooled 1.5-mL microcentrifuge tube and keep on ice.

**Notes**:

- Maintain all samples on ice throughout the procedure and use pre-cooled reagents and equipment to prevent protein degradation. Work quickly throughout the extraction process.
- It is advisable to proceed with protein quantification as soon as possible and avoid freezing the supernatant after lysis to ensure the best results for Atg8a-II detection, as it has been observed repeatedly that freezing between steps results in poorer quality bands. However, if immediate analysis is not possible, the supernatant can be stored at −20°C for later analysis.

### In vivo samples

#### Reagent preparation

**25X Protease inhibitor cocktail, stock solution**: Dissolve one protease inhibitor cocktail tablet (Roche 05,056,489,001) in 2 mL of mqH_2_O to achieve a 25X stock solution. Aliquot 400 µL and store at −20°C.

**1X RIPA lysis buffer stock solution**: Mix 1 mL 10X RIPA (Millipore, 20–188), 400 µL 25X Protease Inhibitor, 8.6 mL of mqH_2_O as well as 1 tablet of Phosphatase Inhibitor Cocktail (Roche 04,906,837,001). Aliquot and store at −20°C.

**1X RIPA lysis buffer with 200 mM NEM, working solution**: Dissolve 0.025 g NEM (Sigma-Aldrich, E3876) (molecular weight = 125.13 g/mol) directly in 1 mL 1X RIPA to reach a final concentration of 200 mM NEM.

**Notes**:

- Due to the high concentration of NEM for *in vivo* samples, there is no need to make a NEM solution stock prior to dissolving in lysis buffer. At this concentration, the amount of NEM to be used can be measured and put directly in the 1X RIPA buffer.
- Due to the high NEM concentration in the lysis buffer, avoid placing the lysis buffer directly into ice to prevent precipitation. Place the tube on top of the ice without pressing it into the ice to keep the lysis buffer cool.

#### Tissue lysis

- After the treatment period, dissect the fat body from approximately 10 *Drosophila* larvae per sample. The dissection is performed on a plate with multiple drops of PBS arranged around a central drop of 200 mM NEM-supplemented lysis buffer.
- In individual PBS droplets, remove the posterior end of the larva with tweezers, invert, and roll to expose internal tissues. Isolate the fat body, ensuring no contamination, and transfer the isolated fat body immediately into the adjacent drop of lysis buffer before dissecting the next larva.
- After collecting all fat bodies, carefully pipette the drop containing the fat bodies and lysis buffer into a pre-cooled 1.5-mL microcentrifuge tube. Keep samples on ice during dissection or store at −20°C for longer dissection series
- Homogenize the samples on ice with a pestle for 5–10 s. Pass the homogenate through a QIAshredder column (Qiagen 79,659) and centrifuge at 18,000 × g for 3 min at 4°C.
- Following this, transfer the lysate to a pre-cooled 1.5-mL microcentrifuge tube and centrifuge at 14,000 × g for 15 min at 4°C. Carefully transfer the supernatant to a new pre-cooled 1.5-mL microcentrifuge tube, ensuring not to disturb the pellet at the bottom of the tube or the lipid layer that form at the top.
- Centrifuge the supernatant again at 14,000 × g for 15 min at 4°C to further clarify, before once more carefully transferring the supernatant to a new pre-cooled 1.5-mL microcentrifuge tube and keep on ice or store at −20°C.

## Protein Quantification Using BCA Assay

### Preparation of BSA standard dilutions

- According to the BCA Protein Assay Kit instructions (Thermo Scientific 23,227), prepare a series of eight dilutions from the BSA stock solution (2 mg/mL) shown in Table 2.

**Table 2.** BSA standard dilution scheme.

Tube	Standard curve	Volume of lysis buffer	Volume of BSA
A	2000 µg/ml	0	300 µL (2 mg/mL stock)
B	1500 µg/ml	125 µL	375 µL (2 mg/mL stock)
C	1000 µg/ml	325 µL	325 µL (2 mg/mL stock)
D	750 µg/ml	175 µL	175 µL (of vial B dilution)
E	500 µg/ml	325 µL	325 µL (of vial C dilution)
F	250 µg/ml	325 µL	325 µL (of vial E dilution)
G	125 µg/ml	325 µL	325 µL (of vial F dilution)
H	25 µg/ml	400 µL	100 µL (of vial G dilution)
I	Lysis buffer only	400 µL	-

### Preparation of BCA working solution

- Prepare the working BCA solution by mixing reagent A and reagent B in a 50:1 ratio, in a total volume for 200 µL for each well. Calculate the total volume of working reagent required using the formula: (number of standards + number of samples) × (number of replicates) × (volume of working reagent per sample) = total volume working reagent required.

### BCA assay procedure and plate reading

- Pipette standard and sample into triplicate wells of a transparent 96-well plate. For optimal results in the 20–2000 µg/mL range, use 25 µL per sample. If sample volume is limited, 5–10 µL can be used, adjusting the range to 125–2000 µg/mL. Ensure all standards and samples are treated consistently if volume adjustments are made.
- Add 200 µL of the BCA working solution to each well and incubate at 37°C for 30 min.
- Allow the plate to equilibrate to room temperature. Read the absorbance at 562 nm using a plate reader.

## Western blot

### Reagent preparation

**Running Buffer (1X)**: Dilute 100 mL 10X Running buffer (0.25 M Tris Base, pH 8.3, 1.92 M glycine, 1.0% w:v SDS) (Bio-Rad 1,610,772) with 900 mL water.

**Transfer Buffer (1X)**: Mix 200 mL 5X Transfer Buffer, (Bio-Rad, 170–4275) with 600 mL distilled water and 200 mL ethanol (100%).

**TBS-Tween 20 (0.1% 1X)**: Combine 100 mL 10X TBS (200 mM Tris, 1500 mM NaCl pH 7.6), 10 mL 10% Tween 20 (Sigma-Aldrich, P1379), and 900 mL distilled water.

### Sample preparation

- Prepare western blot samples according to BCA protein quantification results, by adding 4X NuPage sample buffer (Thermo Fisher, NP0008), 10 × 1 mM DTT (Sigma-Aldrich, D0632) and 5 µg of sample, adjust final volume with water.
- Boil samples at 95°C for 5 min. Allow the samples to equilibrate to room temperature, before spinning them down if condensation has formed on the lid.

### Perform electrophoresis

- Choose appropriate gel, such as 18-well 4–20% Criterion™ TGX™ Precast Gel (Bio-Rad, 567–1094).
- After washing, fill the electrophoresis apparatus with 1X Running Buffer before carefully removing the comb. Wash the wells and load the samples and ladder (Bio-Rad, 161–0374) in the desired order.
- Run at 50-80 V until samples have safely migrated into the gel, then increase to 100-150 V. Continue the run until the dye front reaches the desired position for optimal protein separation.

### Protein transfer

- After electrophoresis, assemble the protein transfer using the Ready-to-Assemble (RTA) Midi LF-PVDF Transfer Kit (Bio-Rad, 170–4275).
- Activate the LF-PVDF membrane by immersing it in ethanol for 30 s, then equilibrate in 1X transfer buffer for 10 min. Soak two stacks of filter paper (Bio-Rad, L002044) in 1X transfer buffer and refrigerate until use to prevent overheating.
- On the anode side of the transfer cassette, layer one stack of soaked filter paper, followed by the LF-PVDF membrane and the gel. Place the second stack of filter paper on top, ensuring full coverage of the gel. Remove air bubbles with a blot roller.
- Secure the cassette lid and insert into the transfer system. Transfer using the semi-dry method (2.5 A/25 V for 7 min). Adjust transfer time for high molecular weight proteins, but avoid overextending transfer to prevent loss of low molecular weight proteins through the membrane.

### Membrane blocking and antibody incubation

- Air-dry the membrane to block nonspecific sites, by hanging it up with a clip at room temperature for a minimum of 10 min.
- Prepare primary antibody solutions, by diluting the primary antibody to working concentration (suggested dilution is specified by the manufacturer) in 1X TBS-Tween 20 with 0.02% NaAzide (G Biosciences, 786–299) and 5% BSA (Sigma-Aldrich, A2153) or skim milk powder (Millipore 70,166).
- Re-activate the dried membrane in 100% ethanol, then wash briefly in TBS-Tween 20.
- Incubate membrane with primary antibodies overnight at 4°C on a rotator or rocking shaker.

### Secondary antibody incubation

- Wash the membranes 3 times for 10 min in TBS-Tween 20 with gentle rocking.
- Incubate with appropriate HRP-conjugated secondary antibodies in 5% BSA or skim milk powder for 1 h at room temperature with gentle rocking.
- Wash the membranes 3 times for 10 min in TBS-Tween 20 with gentle rocking.

### Develop membrane

- Add Supersignal DURA luminol (Thermo Fisher 34,095) to the membrane, and acquire a series of images until saturation of the target band(s) is observed.
- Analyze the images using software such as Image Lab to quantify and interpret results.
